# Monitoring of Tissue Oxygenation: an Everyday Clinical Challenge

**DOI:** 10.3389/fmed.2017.00247

**Published:** 2018-01-16

**Authors:** Zsolt Molnar, Marton Nemeth

**Affiliations:** ^1^Department of Anaesthesiology and Intensive Therapy, University of Szeged, Szeged, Hungary

**Keywords:** venous oxygen saturation, central venous oxygen saturation, oxygen debt, hemodynamic monitoring, oxygen delivery, oxygen consumption, goal-directed therapy

## Abstract

**Purpose of review:**

The aim of this article is to study the overview of pathophysiology and clinical application of central venous oxygen saturation monitoring in critically ill patients and during the perioperative period.

**Recent findings:**

There are several clinical studies and animal experiments evaluating the effects of goal-directed hemodynamic stabilization on critically ill patients. Recent systematic reviews and meta-analyses found that advanced hemodynamic endpoints-targeted management has a positive effect on outcome in high-risk surgical patients. As all interventions aim to improve tissue oxygenation, it is of utmost importance to monitor the balance between oxygen delivery and consumption. For this purpose, central venous blood gas analysis provides an easily available tool in the everyday clinical practice. The adequate interpretation of central venous oxygen saturation renders the need of careful evaluation of several physiological and pathophysiological circumstances. When appropriately evaluated, central venous oxygen saturation can be a valuable component of a multimodal individualized approach, in which components of oxygen delivery are put in the context of the patients’ individual oxygen consumption. In addition to guide therapy, central venous oxygen saturation may also serve as an early warning sign of inadequate oxygen delivery, which would otherwise remain hidden from the attending physician.

**Summary:**

With the incorporation of central venous oxygen saturation in the everyday clinical routine, treatment could be better tailored for the patients’ actual needs; hence, it may also improve outcome.

## Introduction

Interventions to improve oxygen delivery and decrease oxygen consumption are the cornerstone of resuscitation in the critically ill patients and during the perioperative period of high-risk patients. Early recognition of the patients at risks and the implementation of adequate monitoring-guided interventions can have a profound effect on outcome. On the contrary, delaying adequate interventions will inevitably lead to hypoperfusion, tissue hypoxia, and multiple-organ failure affecting both outcome and wasting of resources and costs (1). Therefore, the use of appropriate indices, which are able to detect the imbalance between oxygen delivery (DO_2_) and consumption (VO_2_), is mandatory for adequate management (2). Conventional parameters such as heart rate, mean arterial blood pressure, mental status, and urine output are robust warning signs of inadequate tissue perfusion, but for fine tuning of therapy detailed hemodynamic monitoring is warranted (3). The recent FENICE (Fluid Challenges In Intensive Care) trial indicate that there is a considerable gap between the accumulating knowledge about the benefits of advanced hemodynamic monitoring based optimization and the actual clinical practice. In more than 2,000 patients, fluid challenges were evaluated. The main indicator of administering fluid boluses was hypotension in 57%, and in 43% of cases, no hemodynamic variable was used to predict fluid responsiveness (4). Detailed assessment of global hemodynamic indices such as cardiac output (CO) and derived variables and also the measures of oxygen delivery and uptake should be taken into account to provide appropriate therapy for these patients (5, 6). Furthermore, in addition to the optimization of global hemodynamic parameters, indicators of tissue perfusion should also be monitored to verify the effectiveness of our interventions (7). To monitor changes in tissue oxygenation, central or mixed venous blood gas measurements can give more detailed information, which should be incorporated into a multimodal approach that can lead to a better, individualized, patient-centered care. The goal of this review is to highlight the importance of central venous oxygen saturation in this multimodal, individualized hemodynamic management in the context of the pathophysiological background and the results of recent clinical and experimental studies.

## Physiological Issues

Tissue oxygenation is the net product of oxygen delivery and oxygen consumption, which can be described by the following formulae (8):

$$\begin{array}{l} {\text{DO}}_{2}=\text{CO}\times {\text{CaO}}_{2}.\\ {\text{CaO}}_{2}=\text{Hb}\times 1.34\times {\text{SaO}}_{2}+0.003\times {\text{PaO}}_{2}.\\ {\text{DO}}_{2}=\text{CO}\times (\text{Hb}\times 1.34\times {\text{SaO}}_{2}+0.003\times {\text{PaO}}_{2}\text{)}.\\ {\text{VO}}_{2}=\text{CO}\times {(\text{CaO}}_{2}-{\text{CcvO}}_{2}\text{)}.\\ {\text{VO}}_{2}=\text{CO}\times \text{[(Hb}\times 1.34\times {\text{SaO}}_{2}+0.003\times {\text{PaO}}_{2}\text{)}\\ \;-(\text{Hb}\times 1.34\times {\text{ScvO}}_{2}+0.003\times {\text{PcvO}}_{2})].\\ \text{Oxygen extraction }\left({\text{O}}_{2}\text{ER}\right)={\text{VO}}_{2}{\text{/DO}}_{2}.\\ {\text{O}}_{2}\text{ER: }\left({\text{SaO}}_{2}-{\text{ScvO}}_{2}\right){\text{/SaO}}_{2}. \end{array}$$

If SaO_2_ is taken as 1, as under normal circumstances, the hemoglobin is almost fully saturated with oxygen, and the other hemodynamic variables are kept constant, then:

$${\text{O}}_{2}\text{ER}\approx \text{1 }-{\text{ ScvO}}_{2}$$

DO_2_, oxygen delivery; CO, cardiac output; Hb, hemoglobin; SaO_2_, arterial oxygen saturation; PaO_2_, partial pressure of oxygen in the arterial blood; CaO_2_, arterial oxygen content; VO_2_, oxygen consumption; ScvO_2_, central venous oxygen saturation; CcvO_2_, central venous oxygen content; O_2_ER, oxygen extraction; PcvO_2_, central venous partial pressure of oxygen.

Taking a 75-kg healthy adult man when resting, the relationship between DO_2_ and VO_2_ can be estimated as:

Oxygen delivery:

$$\begin{array}{l} \text{CO}=70\text{ml}\times 70/\text{min}\sim 5,000\text{ ml}/\text{min}.\\ {\text{CaO}}_{2}=\left(150\text{g}/\text{l }\times 1.34\text{ ml}\times \text{ 1}.00\right)+(0.003\times 100\text{ mmHg})\sim 200\text{ ml}/l.\\ {\text{DO}}_{2}\;\sim \;1,000\text{ ml}/\text{min}. \end{array}$$

Oxygen consumption:

$$\begin{array}{l} \text{CO}=\text{70ml }\times \text{ 70/min \textasciitilde{} 5,000 ml/min}.\\ {\text{CcvO}}_{2}=(150\text{ g}/\text{l}\times 1\text{.34 ml}\times 0\text{.75) + (0}.003\times 40\text{ mmHg) \textasciitilde{} 150 ml/l}\\ {\text{VO}}_{2}=\text{5l/min}\times \;\left(200\text{ ml/l }-\text{ 150 ml/l}\right)\text{ \textasciitilde{} 250 ml/min}. \end{array}$$

Oxygen extraction:

$${\text{O}}_{2}\text{ER: 250 ml/min/1,000 ml/min }\times \text{ 100}=25\%.$$

The main difference between the equations of DO_2_ and VO_2_ is the oxygen content (CaO_2_ versus CcvO_2_), especially the central venous oxygen saturation (ScvO_2_). Therefore, it can be useful to assess the imbalance between DO_2_ and VO_2_ in the critically ill.

**Figure 1:**
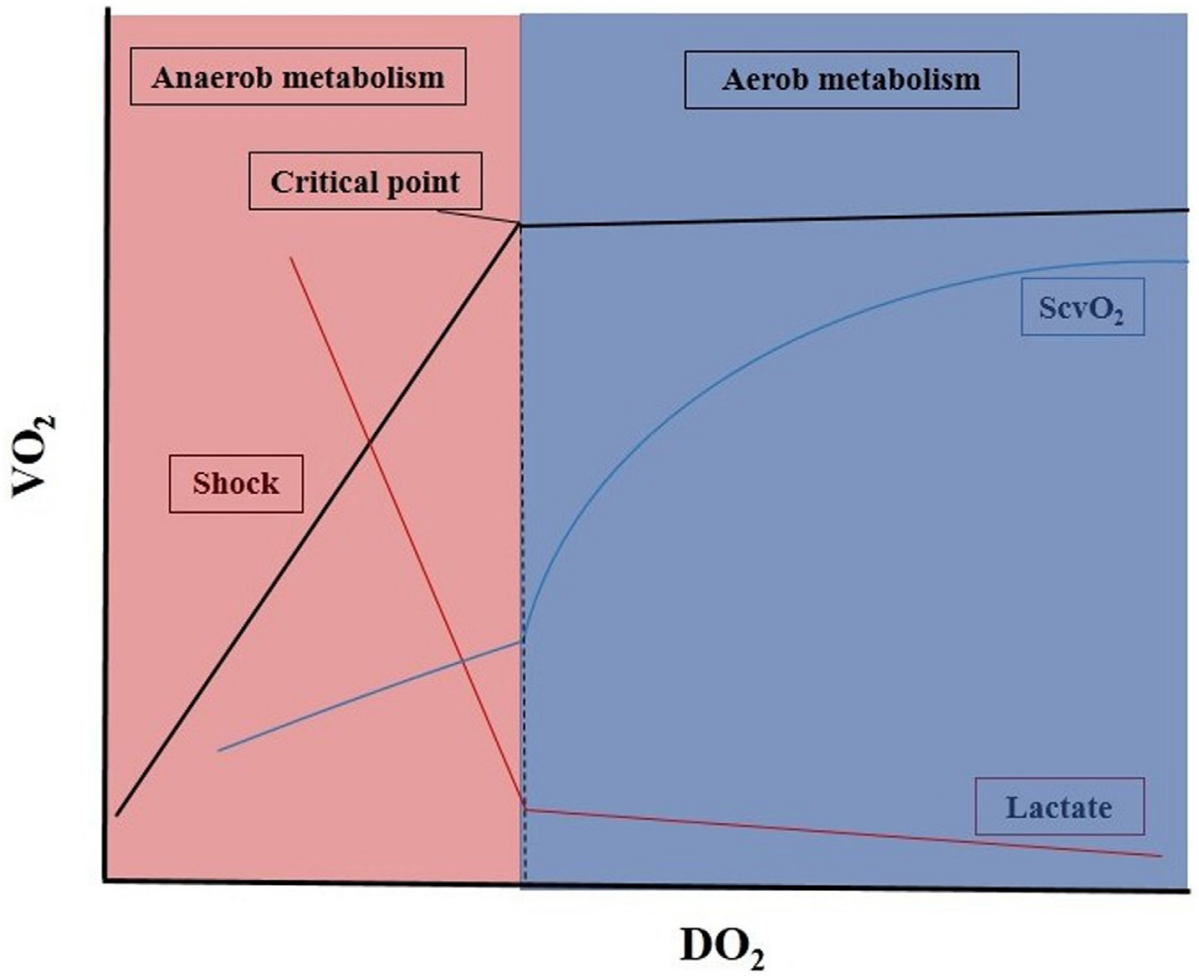
Oxygen delivery and consumption in critically ill patients. DO_2_, oxygen delivery; VO_2_, oxygen consumption; ScvO_2_, central venous oxygen saturation ratio. For details, see main text.

When the arterial oxygen content (CaO_2_) and/or CO becomes impaired, DO_2_ decreases, which is often accompanied by a parallel decrease in VO_2_. The most frequently occurring scenarios are represented in Figure 1. In the early phase of decreasing DO_2_, the circulation can compensate to some extent, and VO_2_ remains stable. However, beyond a critical point, any further drop in DO_2_ will result in a decrease in VO_2_. From this point, VO_2_ becomes dependent on DO_2_, and aerobic metabolism will have to be switched to anaerobic metabolism, leading to low ScvO_2_, hyperlactatemia, metabolic acidosis, and oxygen debt (9).

The principle task of early resuscitation is to regain balance by optimizing the VO_2_/DO_2_ ratio. However, it is also important to define the endpoints of resuscitation to avoid overresuscitation. In the case of fluid resuscitation, for example, unnecessary administration of fluids will lead to hypervolemia, which increases morbidity and mortality to a similar extent to that of hypovolemia (10, 11). Unjustified blood transfusions also carry the risk of hypervolemia and transmission of infections (12) or allergic reactions (13). There is evidence that prolonged use of catecholamines is associated with poor outcome (14). Therefore, it is important to recognize the point when tissue perfusion has been normalized, oxygen debt has been resolved, and resuscitation has been terminated.

## Individualized Goal-Directed Hemodynamic Therapy

The multimodal concept in hemodynamic monitoring enables us to appreciate that each patient is different, hence the so-called normal values, which are more or less appropriate for a given population may be inadequate for the given patient. Therefore, this concept can be translated into the individualized or personalized use of target endpoints to avoid underresuscitation or overresuscitation.

## Parameters for Assessment of Tissue Metabolism

### Mixed Venous and Central Venous Oxygen Saturation

Mixed venous oxygen saturation (SvO_2_) measured in the pulmonary artery *via* a pulmonary artery catheter, and its surrogate, central venous oxygen saturation (ScvO_2_) measured in the superior vena cava are the most commonly used parameters to assess global oxygen extraction (VO_2_/DO_2_). As central venous catheters are frequently applied in most critically ill patients, ScvO_2_ is more readily available compared to SvO_2_. Although the absolute values of ScvO_2_ are 5% higher than SvO_2_ on average, but changes usually occur in a parallel manner (15), therefore ScvO_2_ is regarded as a surrogate marker in the clinical setting (16, 17).

The main factors, which influence ScvO_2_, are hemoglobin, arterial oxygen saturation of hemoglobin, CO, and oxygen consumption. There are multiple physiologic, pathophysiologic, and therapeutic factors that influence venous oxygen saturation such as anemia, hypovolemia, contractility, bleeding, sedation, fever, and pain (18).

### ScvO_2_ in Intensive Care Patients

During sepsis, organ dysfunction is most likely the result of inadequate tissue perfusion causing cellular hypoxia. Interventions improving the balance between DO_2_ and VO_2_ may prevent the development of tissue hypoperfusion, organ dysfunction syndrome, and thus improve the outcome of septic patients. In patients with early phase of severe sepsis, septic shock, early goal-directed intervention guided by continuous monitoring of ScvO_2_, central venous pressure and mean arterial pressure (MAP), with target values of CVP 8 to 12 mmHg, MAP > 65 mmHg and ScvO_2_ > 70%, reduced mortality from 46.5 to 30.5% at the 28th day (19).

Although this study has been criticized for several reasons and these results could never be repeated, there is international consensus that that low ScvO_2_ values are very important warning signs of inadequate DO_2_ and can prognosticate complications and poor outcome. However, recent data suggest that high ScvO_2_ values may also have adverse outcomes in septic patients (20). Due to deranged microcirculation when shunting is present on the level of capillaries, impaired oxygen utilization can lead to normal or supraphysiological ScvO_2_ values, which represent an inability of the cells to extract oxygen in sepsis (21). In patients with ScvO_2_ > 70% complementary blood gas parameters, such as elevated venous-to-arterial CO_2_ gap (dCO_2_) (>6 mmHg), increased or persistently elevated serum lactate levels could help the clinicians to identify tissue hypoxia. In a retrospective analysis, septic patients with physiological ScvO_2_ and abnormal dCO_2_ mortality was significantly higher as compared to patients with normal dCO_2_ values (22).

In patients treated on intensive care units, heart failure is often present resulting impaired CO, hence decreased oxygen delivery (23), and resulting oxygen extraction imbalance that could be detected by low ScvO_2_ (24). In a clinical study after myocardial infarction in patients with heart failure and cardiogenic shock, SvO_2_ was 43%, while in patients with heart failure without shock, it was 56% compared to patients without heart failure with an SvO_2_ of 70% (25). It may also be useful in patients with cardiogenic shock requiring the support by intraaortic balloon counter pulsation. In a study during weaning period, intraaortic balloon pump assist ratio was decreased from 1:1 to 1:3. In the weaning failure group, decreased support was accompanied by a drop in ScvO_2_, while it remained constant in the successful group (26). In patients with chronic heart failure, ScvO_2_ can be chronically low. However, during acute decompensation, major cardiac events were observed in 81% of patients with ScvO_2_ ≤ 60% at 24 h after ICU admission, while it was only 13% in patients with higher ScvO_2_ (27).

### ScvO_2_ and Blood Transfusion

In addition to heart failure, anemia is another frequent cause of impaired DO_2_ in critically ill patients, and almost 40–45% of patients will receive blood transfusion during the treatment period (28). As large multicenter trials (TRICC and TRISS) suggest that patients with hemoglobin levels above 10 mg/dl usually do not require transfusion, while red blood cell administration is usually beneficial if the hemoglobin level is below 7 mg/dl (29, 30). Between these values, physicians have to make decisions according to clinical signs like mental status, tachycardia, tachypnea, blood pressure, and diuresis. To be able to give additional objective data about oxygen debt of organs, ScvO_2_ may offer an easily obtainable tool to detect a low hemoglobin-related altered O_2_ER and hence may serve as a physiological trigger for blood transfusion (30). In human studies, both on volunteers and retrospective data in critically ill patients suggest that lower levels of hemoglobin compared to that of recommended by international guidelines were well tolerated and did not produce hemodynamic instability, and when oxygen imbalance occurred, it was accompanied by a significant drop in SvO_2_ (30–32). In our recent animal experiment on isovolemic anemia, we have found that anemia-induced change in VO_2_/DO_2_ showed significant correlation with changes of ScvO_2_ (33); hence, ScvO_2_ may be used as a “physiologic transfusion trigger” in otherwise hemodynamically stable patients.

### ScvO_2_ and High-Risk Surgery

High-risk surgical patients are at an increased risk of developing imbalance between VO_2_ and DO_2_ in the perioperative period; therefore, monitoring ScvO_2_ may have a rationale during both the intraoperative and postoperative managements.

It has been shown that patients with low ScvO_2_ values preoperatively, intraoperatively, or postoperatively are at an increased risk for complications and poor prognosis (34). Therefore, it seems to be logical to maintain ScvO_2_ in normal range during the perioperative care. We reported in a small, single-center prospective randomized study about continuously measured ScvO_2_-assisted intraoperative hemodynamic optimization (CeVOX Maquet^®^ Munich Germany) during major abdominal surgery. In the conventional group, patients were treated according to mean arterial and central venous pressure, while in the ScvO_2_ group, additionally venous oxygen saturation was also measured *via* fiberoptic catheter placed in the superior vena cava. ScvO_2_ monitorization resulted in more interventions, more fluid boluses and more blood transfusion compared to the conventional group. These intervention resulted in better organ functions, less complication rate, and better 28 days of survival (35). These results are in accord with the results of an earlier single-center study, where ScvO_2_ over 73% directed group had fewer postoperative complications and had shorter length of hospital stay compared to patients in whom hemodynamic stabilization was guided according to MAP and central venous pressure (36). However, it is important to considerate that in anesthetized, mechanically ventilated patients, “physiological” values of ScvO_2_ are 5–10% higher (i.e., 75–80%) because of the decreased oxygen extraction of the brain. Second, when bleeding is present and blood loss is replaced by crystalloids, considerable hemodilution can take place. In our experimental stroke volume-guided hemorrhage and fluid resuscitation animal model, ScvO_2_ normalized at the end of resuscitation, but returned to a significantly lower level (with a mean of 5%) as the hemodilution caused significant drop in hemoglobin levels (37). In a clinical study performed on patients with esophagectomy, ScvO_2_ could indicate decreased DO_2_ caused by low hemoglobin levels; therefore, the authors suggest to use ScvO_2_ as complementary transfusion trigger to hemoglobin in the perioperative period (32).

High-risk patient with major surgery benefits most from goal-directed therapy with significant reduction in mortality and morbidity compared to patients with low-risk interventions (38). ScvO_2_ is an important element of this complex perioperative multimodal monitoring-based concept, including advanced hemodynamic monitoring and assessment of VO_2_/DO_2_, what we call the individualized, multimodal approach (39).

## Complementary Blood Gas Parameters

### Venous-to-Arterial CO_2_ Gap (dCO_2_)

Mixed-, or central venous-to-arterial carbon dioxide gap is an easily attainable parameter when patients has arterial and central venous lines *in situ*. The physiological value is ≤6 mmHg, and this holds true for both mixed- (Pv-aCO_2_) and central venous-to-arterial (Pcv-aCO_2_) CO_2_ gap values. Therefore, the central venous Pcv-aCO_2_-gap can be useful surrogate of Pv-aCO_2_ in the everyday practice.

Increased CO_2_ gap of >40 mmHg was described 30 years ago during cardiac arrest in patients who were monitored with pulmonary artery catheters and also in an animal experiment on cardiopulmonary resuscitation (40). After these landmark studies, increased dCO_2_ was detected in several low-flow states (41–43). During anaerobic metabolism, increased production of hydrogen ions are buffered by bicarbonate presented in the cells, and this process will generate CO_2_ production (44). When the Fick principle is applied for carbon dioxide, there is an inverse relationship between the CO and dCO_2_ (45); in other words, increased levels of dCO_2_ should reflect low-flow states. Indeed, it has been shown that in sepsis, heart failure, and severe hypovolemia, its value can be elevated (46, 47).

In the perioperative setting, dCO_2_ also has a strong predictive value. Patients with high dCO_2_ had significantly higher mortality compared to patients with normal values (36.4 versus 4.5%) (48). High-risk surgical patients admitted to intensive care unit postoperatively with high dCO_2_ also developed more complications. The cutoff value was 5.8 mmHg (49), and in a different clinical study, a dCO_2_ > 5 mmHg had 96% sensitivity to predict the occurrence of postoperative complications in patients with physiological (≥71%) ScvO_2_ (50). In critically ill patients, the dCO_2_ shows good inverse correlation with the CO (42), and it has also been shown to be a good predictor for bad outcome in patients with septic shock (41). In cases like septic shock, when due to microcirculatory or mitochondrial defects oxygen uptake is insufficient, ScvO_2_ can be supranormal. Previous studies have suggested that under such circumstances the increased value of dCO_2_ (>5 mmHg) and increased lactate level can help the physician in detecting inadequate flow to the tissues; hence, the complementary use of ScvO_2_ and dCO_2_ is recommended (50, 51).

## Conclusion

Early and adequate interventions to improve hemodynamics, oxygen delivery, and reducing oxygen needs have a significant effect on outcome. Protocolized care with predefined values of certain physiological indices, such as blood pressure, CO, may benefit the majority of the population, but these values may be inadequate for the rest; hence, they will remain either underresuscitated or overresuscitated. Therefore, individualizing treatment should be desirable. For this purpose, additional physiological parameters like central venous oxygen saturation, lactate, and venous-to-arterial CO_2_ gap should be assessed together with other hemodynamic variables to get a detailed picture about the hemodynamic status of our patients. Putting the pieces of the puzzle together in context is what we define as multimodal, individualized hemodynamic support, in which ScvO_2_ has a pivotal role.

## Author Contributions

All authors listed have made a substantial, direct and intellectual contribution to the work, and approved it for publication.

## Conflict of Interest Statement

ZM receives regular honoraria for being in the Medical Advisory Board of PULSION Maquet, for lectures from Biotest, ThermoFisher Scientific, and CytoSorbents. The remaining author declares that the research was conducted in the absence of any commercial or financial relationships that could be construed as a potential conflict of interest.
